# Virotherapy Using Myxoma Virus Prevents Lethal Graft-versus-Host Disease following Xeno-Transplantation with Primary Human Hematopoietic Stem Cells

**DOI:** 10.1371/journal.pone.0043298

**Published:** 2012-08-14

**Authors:** Eric Bartee, Amy Meacham, Elizabeth Wise, Christopher R. Cogle, Grant McFadden

**Affiliations:** 1 Department of Molecular Genetics and Microbiology, University of Florida, College of Medicine, Gainesville, Florida, United States of America; 2 Division of Hematology/Oncology, Department of Medicine, University of Florida, College of Medicine, Gainesville, Florida, United States of America; Public Health Agency of Canada, Canada

## Abstract

Graft-versus-host disease (GVHD) is a potentially lethal clinical complication arising from the transfer of alloreactive T lymphocytes into immunocompromised recipients. Despite conventional methods of T cell depletion, GVHD remains a major challenge in allogeneic hematopoietic cell transplant. Here, we demonstrate a novel method of preventing GVHD by *ex vivo* treatment of primary human hematopoietic cell sources with myxoma virus, a rabbit specific poxvirus currently under development for oncolytic virotherapy. This pretreatment dramatically increases post-transplant survival of immunocompromised mice injected with primary human bone marrow or peripheral blood cells and prevents the expansion of human CD3^+^ lymphocytes in major recipient organs. Similar viral treatment also prevents human-human mixed alloreactive T lymphocyte reactions *in vitro*. Our data suggest that *ex vivo* virotherapy with myxoma virus can be a simple and effective method for preventing GVHD following infusion of hematopoietic products containing alloreactive T lymphocytes such as: allogeneic hematopoietic stem and progenitor cells, donor leukocyte infusions and blood transfusions.

## Introduction

Graft-versus-host disease (GVHD) affects approximately 25–50% of patients receiving allogeneic hematopoietic cell transplantation (alloHCT), of which 15% will die [1], [2]. In addition, GVHD occurs in approximately 1% of patients receiving high-risk blood transfusions where it is often fatal [3]. One major cause of GVHD is the transfer of mature donor CD3^+^ T lymphocytes into an immunocompromised recipient. Once infused, a subset of alloreactive T lymphocytes recognizes recipient cellular antigens and undergo activation and amplification, resulting in an severe immunoreactive cascade which affects many internal organs, particularly the liver, gastrointestinal tract and skin [4], [5].

Current methods to prevent and treat GVHD include: general immune suppression following transplant, reduced intensity conditioning, and depletion or inhibition of alloreactive donor CD3^+^ lymphocytes prior to transfusion [6], [7], . The clinical effectiveness of these methods, however, is limited. For example, general immune suppression leads to an increased risk of viral reactivation and other opportunistic infections, while reduced intensity conditioning regimens are associated with increased relapse [7]. Currently, the most promising prophylactic treatment for GVHD appears to be depletion or inhibition of alloreactive donor T lymphocytes prior to infusion [11], [12]. This can be accomplished through a variety of methods, including lymphoablative cytotoxic agents, specific T lymphocyte inhibitors, and antibody based selections. Unfortunately, while these methods have proven effective at lowering the rates of GVHD; they are also associated with slower reconstitution of the recipient immune system, increased risk for life-threatening infections, and reductions in the beneficial graft-versus-leukemia (GVL) effect [13], [14]. There is currently no effective treatment for GVHD resulting from high risk blood transfusions [3]. Due to its frequency and severity, GVHD is currently the major factor limiting the use of alloHCT and therefore represents a significant, unresolved clinical issue for the treatment of a wide variety of diseases, including leukemias, lymphomas, bone marrow failure syndromes, and autoimmune diseases. Novel strategies to mitigate GVHD, particularly strategies that maintain the beneficial GVL effect, are therefore needed to advance transplant and transfusion technology, especially in higher risk transplant regimens such as haploidentical transplant where risks of GVHD are extremely high.

Our lab has recently demonstrated that the rabbit specific poxvirus, myxoma virus (MYXV), can be used as a novel *ex vivo* purging agent to functionally eliminate specific malignant cell populations from human HCT samples [15], [16], [17], [18]. MYXV has several advantages as a virotherapeutic in humans. First, MYXV's natural host range is tightly restricted to rabbits and no instances of MYXV infection have been documented in any non-rabbit species, even following injection of live virus into human subjects or immunocompromised mice [19], [20]. Second, MYXV binding is thought to depend on relatively ubiquitous glycosaminoglycans at the cell surface rather than specific protein entry receptors making this virus a good candidate for treating a wide variety of human cancers. In contrast, one notable cell type that myxoma virus cannot either bind or infect is normal human CD34^+^ hematopoietic stem and progenitor cells (HSPCs), thus MYXV treatment does not alter the efficient engraftment of this cell population into immunodeficient mice [15], [16], [17]. Finally, the *ex vivo* application of MYXV to hematopoietic products is relatively quick and uncomplicated, making it an attractive adjunct therapy for clinical administration.

During our ongoing studies to optimize the purging of contaminating cancer cells from normal human stem cell samples, we unexpectedly observed that immunodeficient mice transplanted with MYXV-treated human hematopoietic products displayed significantly prolonged survival times compared to mock-treated transplant controls. To better understand this observation, we examined the ability of *ex vivo* MYXV treatment to functionally eliminate GVHD-inducing T lymphocytes from human alloHCT samples. Our data shows that MYXV infects a small subset of primary human CD3^+^ lymphocytes found in both bone marrow and peripheral blood derived HCT samples. Additionally, *ex vivo* treatment of these HCT samples with MXYV delayed or eliminated development of lethal GVHD in xeno-transplanted mice, while fully preserving GVL. Our results indicate that *ex vivo* treatment of HCT samples with MYXV prior to infusion shows promise as a novel method to clinically prevent development of GHVD following alloHCT.

## Materials and Methods

### Normal Human Cells

Fresh normal human bone marrow aspirate cells and peripheral blood mononuclear cells were obtained commercially from Lonza (Walkersville, Maryland). Bone marrow mononuclear cells (BM-MNCs) were then enriched over a Ficoll gradient using a clinical Sepax device (Biosafe Inc.) as per manufacturer's recommendations.

### Myxoma virus and viral Infections

MYXV infections were carried out by incubating primary human cells with vMyx-GFP (a MYXV construct which expresses eGFP at an intergenic location in the viral genome from a synthetic viral early/late promoter [21]). Human BM-MNCs were exposed to virus at a multiplicity of infection (MOI) of 10 for 1 hour in PBS + 10% FBS in a humidified chamber at 37°C and 5% CO_2_. Mock treated cells were incubated in PBS plus 10% FBS containing no virus under the same incubation conditions.

### Human Stem Cell Xenografts in NSG mice

For GVHD studies, NOD/Scid/IL2Rγ^−/−^ (NSG) mice were sublethally irradiated using 175 cGy total body irradiation from a Cs^137^ source. Within twenty-four hours after irradiation, mice were injected through the tail vein with 1×10^6^ – 10×10^6^ primary human BM-MNCs or peripheral blood mononuclear cells (PBMCs) that had been either mock treated or treated with vMyx-GFP. Prophylactic antibiotics were administered in the drinking water for two weeks after transplantation to prevent opportunistic bacterial infection. Mice showing evidence of post-transplant disease were sacrificed if they reached a body condition score of 2 according to [22] in accordance with approved University of Florida IACUC protocol 201105023. Surviving mice were euthanized six weeks after transplantation and bone marrow was harvested. Human hematopoietic cell engraftment was quantified using flow cytometry (BD FACSCaliber) for human CD45^+^ and HLA-A, B, C^+^ cells. Mice were scored as engrafted if flow cytometry confirmed populations of cells present in bone marrow that were human CD45^+^/HLA-A, B, C^+^ double positive. The number of CD45^+^/HLA-A, B, C^+^ cells in each bone marrow sample is presented as level of engraftment. Lineage analysis of engrafted cells was determined by co-staining extracted murine bone marrow with the following antibodies: HLA-APC, CD3-PE, CD19-FitC, CD15-PerCP.

### Immunohistochemistry

Analysis of infiltration of human CD3^+^ lymphocytes into murine peripheral tissues was accomplished by surgically removing six tissues post mortem: lung, liver, kidney, spleen, skin, and intestine. Tissues were fixed in 10% formalin buffered with PBS for 24 hours and then washed in 70% ethanol for an additional 24 hours. Five-micron sections of formalin-fixed, paraffin-embedded blocks were cut and picked-up onto plus charged slides (Fisher Scientific). Slides were deparaffinized and rehydrated through a series of xylenes and graded alcohols and blocked in 3% peroxide/methanol for 10 minutes at room temperature. Heat mediated antigen retrieval was performed in Citra buffer pH 6.0 for 25 minutes. This was immediately followed by blocking with normal goat serum and avidin/biotin using a commercially available kit (Vector Labs). Rabbit anti-huCD3 was applied to the sections at 1∶100 overnight at 4°C. Staining was completed using an ABC-Elite kit (Vector Laboratories). The antigen-antibody complex was observed by reaction with DAB (Vector Labs) and slides were counterstained with hematoxylin prior to coverslipping.

### Magnetic bead cell sorting

CD3^+^ and CD34^+^ human cells were fractionated from primary human BM-MNCs using the CD3^+^ (Cat#130-050-101) and CD34^+^ (Cat#130-046-702) microbead separation kits from Miltenyi Biotec as per manufacturer's recommendations. Cells were then positively selected on an autoMACS pro separator (Miltenyi Biotec) as per manufacturer's recommendations. The relative purity of each fractionated population was confirmed after separation using flow cytometry. The total number of fractionated cells was determined after separation using a hemocytometer.

### Human:Human Mixed Lymphocyte Reaction Assays

1×10^6^ SEPAX purified human BM cells were plated in triplicate into each well of a 96-well plate. Cells were then irradiated using 1000 cGy from a Cs^137^ source to create replication incompetent feeder cells. BM-MNCs cells from a second HLA-mismatched donor were either mock-treated or treated with MYXV and then 1×10^6^ cells were plated in triplicate into empty wells or wells containing irradiated feeder cells. At the indicated time points, the total number of viable cells in each well was determined using a commercial MTT assay (Pierce) as per the manufacturer's recommendations.

### Graft-versus-Leukemia Studies

To pre-establish cancer niches in the bone marrow of mice prior to transplant of human HCT samples, non-irradiated NSG mice were injected through the tail vein with 1×10^6^ human multiple myeloma cells (U266 cell line expressing the HLA-A2.1 haplotype). Two weeks after injection of the myeloma cells, mice were sublethally irradiated and injected with 10×10^6^ human BM-MNCs cells that had been either mock treated or treated with vMyx-GFP as described above. Six weeks after transplant of human BM-MNCs, surviving mice were euthanized and bone marrow was harvested. Levels of human hematopoietic cell engraftment and pre-existing U266 myeloma load were distinguished and quantified using flow cytometry (BD FACSCaliber). Cells were distinguished based on expression of HLA-A, B, C, HLA-A2.1 and CD45 (hematopoietic stem cell progeny are HLA-A, B, C^+^/HLA-A2.1^−^/CD45^+^ while U266 myeloma cells are HLA-A, B, C^+^/HLA-A2.1^+^/CD45^−^).

### Statistical Analysis

Statistical differences between different experimental groups were determined by log-rank and Student's t-test. The reported values represent the mean plus or minus the standard error of the mean. A *P* value of less than 0.05 was considered statistically significant.

### Ethic Statement

All animal work in this study was carried out in strict accordance with the recommendations in the Guide for the Care and Use of Laboratory Animals of the National Institutes of Health. The protocol was approved by the University of Florida Institutional Animal Care and Use Committee (Protocol Number: 201105023).

## Results

### MYXV binds and infects a subset of CD3^+^ cells

**Figure 1:**
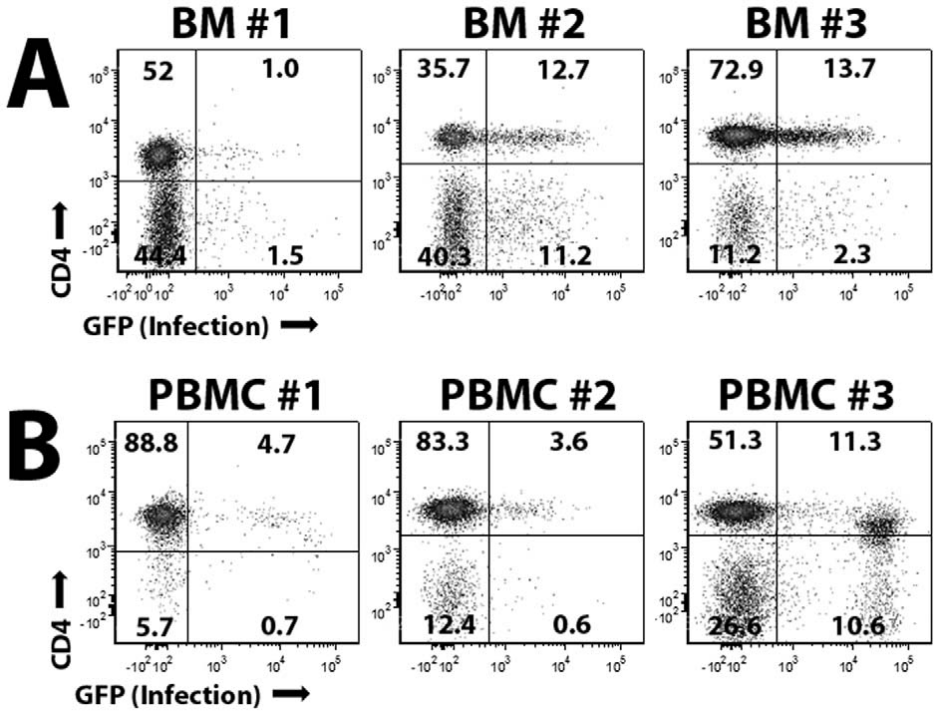
MYXV infects a subset of primary human CD3^+^ lymphocytes. To determine if MYXV can infect CD3^+^ lymphocytes found in human HCT samples, 1×10^6^ whole BM cells (**A**) or 1×10^6^ human PBMCs (**B**) were treated with vMyx-GFP at MOI = 10. Twenty-four hours after MYXV exposure, cells were stained with antibodies against CD3 and CD4 and the levels of GFP^+^ cells in each population was determined using flow cytometry.

Chahroudi et al have previously demonstrated that vaccinia virus, a related poxvirus from a distinct genus, infects a subset of CD3^+^ lymphocytes found in human peripheral blood [23]. To determine whether MYXV might also infect CD3^+^ lymphocytes found in HCT samples, primary human BM-MNCs (Figure 1A) or primary human PBMCs (Figure 1B) were incubated with vMyx-GFP at MOI = 10. After 24 hours, cells were washed, stained with anti-CD3, anti-CD4 and anti-CD8 antibodies, and analyzed using flow cytometry. Successful infection of lymphocytes was readily detected by expression of GFP encoded by vMyx-GFP. Consistent with previous reports for vaccinia virus [23], MYXV infection was observed in a small subset of CD3^+^ lymphocytes ranging from 1% to 15%, depending on the patient. No significant preference was observed for infection of CD4^+^ vs CD8^+^ lymphocytes in individual patient samples (data not shown).

### MYXV treatment prevents lethal GVHD following xenotransplant of human BM-MNCs

**Figure 2:**
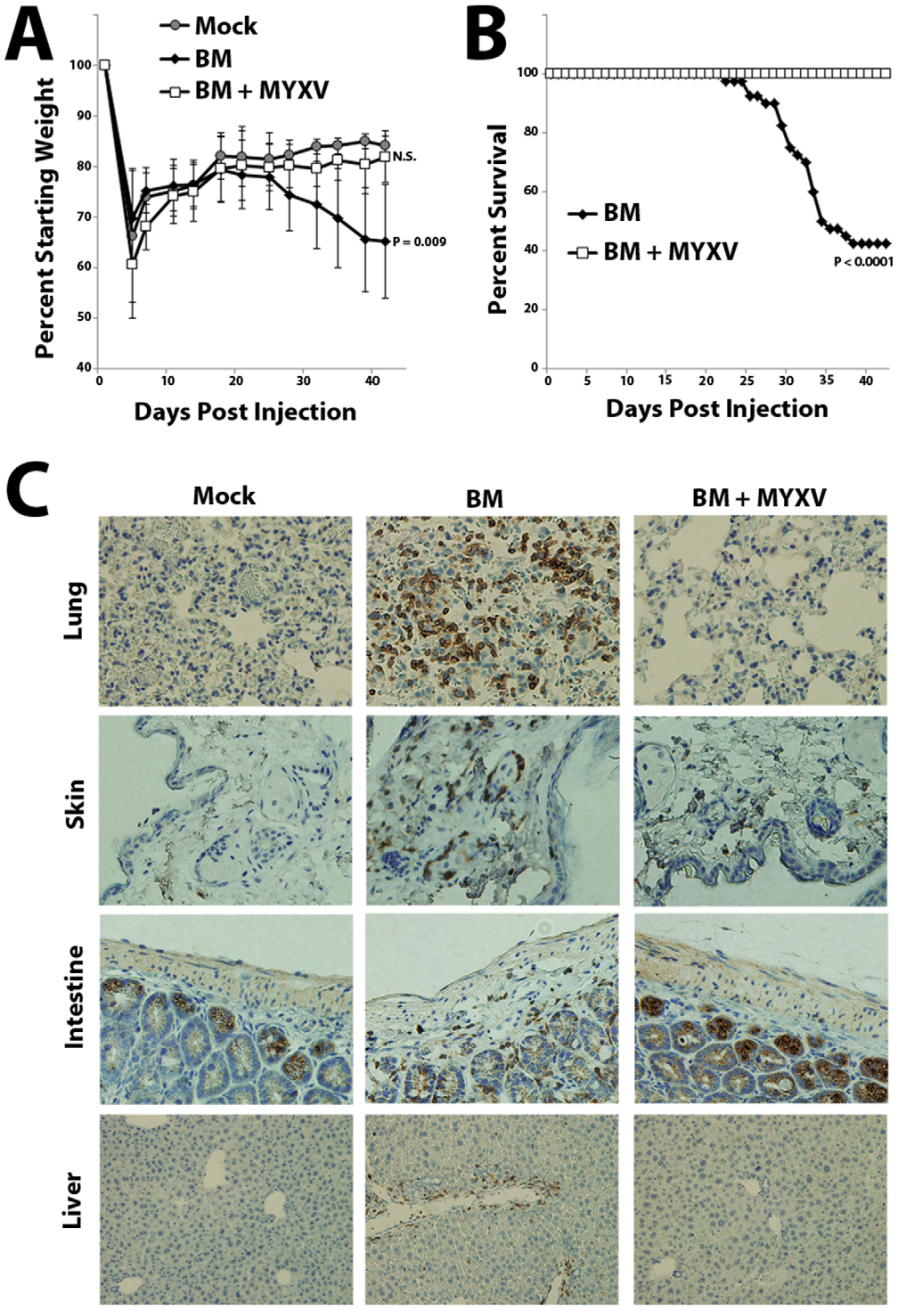
MYXV-treatment prevents lethal GVHD in immunodeficient mice following infusion of primary human BM. NSG mice were sublethally irradiated and then transplanted with either PBS (mock, n = 5), 1×10^7^ primary human BM (n = 36) or 1×10^7^ primary human BM pre-treated with MYXV (n = 36). Mice were weighed twice per week to monitor body condition (**A**) and sacrificed either six weeks after transplant or when their body condition score measured 2 (**B**). Significant differences in survival were determined using the log-rank test (P<0.05). N.S.  =  not significant. Post-mortem, organs were extracted, fixed in formalin, sectioned and stained for the presence of infiltrating human CD3^+^ lymphocytes (**C**). Immunohistochemistry images shown are representative of results observed in five separate mice from the three engrafted cohorts stained for the presence of human CD3^+^ cells.

**Figure 3:**
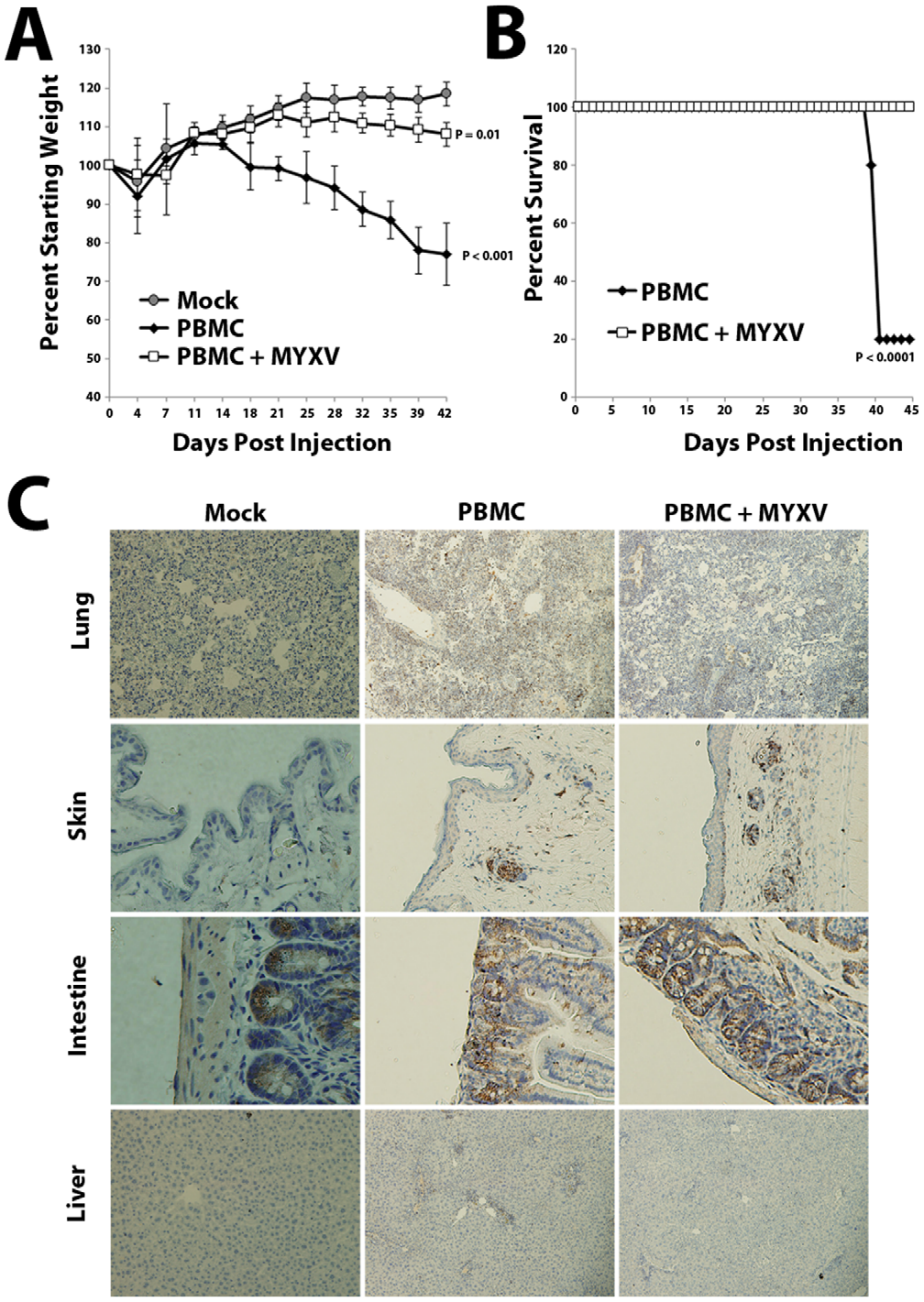
MYXV-treatment prevents lethal GVHD in immunodeficient mice following infusion of primary human PBMCs. NSG mice were sublethally irradiated and then transplanted with either 5×10^6^ primary human PBMCs (n = 6) or 5×10^6^ primary human PBMCs pre-treated with MYXV (n = 6). Mice were weighed twice per week to monitor body condition (**A**) and sacrificed either seven weeks after transplant or when their body condition score measured 2 (**B**). Significant differences in survival were determined using the log-rank test (P<0.05). N.S.  =  not significant. Post-mortem, organs were extracted, fixed in formalin, sectioned and stained for the presence of human CD3^+^ lymphocytes (**C**). Immunohistochemistry images shown are representative of results observed in three separate mice from the three engrafted cohorts stained for the presence of human CD3^+^ cells.

A previous report indicated that vaccinia virus preferentially infects mature CD3^+^ lymphocytes, compared to naïve T cells [23]. Since mature CD3^+^ T lymphocytes are thought to be a major driver of GVHD, we wanted to examine whether treatment with MYXV could prevent development of GVHD in a murine model of allo-HCT. We therefore injected sublethally irradiated NSG mice with either normal human BM-MNCs or PBMCs, which are known to cause a highly lethal form of xeno-GVHD [24]. In our study, 50–70% of sub-lethally irradiated NSG mice transplanted with healthy human BM-MNCs developed a lethal wasting disease by 4–6 weeks after transplant (Figure 2A and 2B). This disease was not observed in irradiated mock-injected control mice or in irradiated mice injected with established cancer cell lines but was consistently observed following injection of primary BM obtained from three distinct healthy donors (Figures S1 and Table S1). Similar results were observed in mice infused with human PBMCs from two distinct donors (Figure 3). Consistent with these mice developing GVHD, post-mortem histology of diseased mice revealed significant edema as well as infiltration of human CD3^+^ T lymphocytes into several organs, including the liver, intestines, skin, lung, kidney and spleen (Figures 2C and S1). In sharp contrast, mice injected with normal human BM or PBMCs which had been pretreated *ex vivo* with MYXV universally survived without evidence for any obvious disease (Figures 2 and 3). Additional post-mortem histology revealed that mice injected with MYXV-treated BM displayed virtually no infiltration of human CD3^+^ T lymphocytes into any major internal organ (Figures 2C and S1). These data suggest that *ex vivo* pretreatment of allo-HCT samples with MYXV prior to transplant might prevent the subsequent development of GVHD.

### Xeno-GVHD results from transfusion of human CD3^+^ lymphocytes

Our *in vivo* experiments indicate that MYXV can prevent GVHD after xeno-transplant of either human BM-MNCs or PBMCs. While allo-reactive donor CD3^+^ lymphocytes are the primary cause of human GVHD, other cell types, such as NK cells or CD34^+^, might play a role in the development of GVHD in the murine xeno-transplant model. We therefore wished to confirm that CD3^+^ lymphocytes were the population causing disease in our xeno-transplant model. To accomplish this, we used immunomagnetic enrichment or depletion of CD3^+^ T lymphocytes or CD34^+^ HSPC from primary human BM. Consistent with T lymphocyte-mediated acute GVHD, mice xenotransplanted with either positively selected CD3^+^ lymphocytes or BM depleted of CD34^+^ cells succumbed to GVHD with kinetics similar to mice transplanted with unfractionationed BM (Table S1). In contrast, mice transplanted with BM depleted of CD3+ lymphocytes or positively selected CD34+ HSPCs failed to develop GVHD (Table S1). In all cohorts, NSG mice transplanted with enriched or depleted human cells pretreated with MYXV universally survived and presented no evidence of GVHD (Table S1).

### MYXV prevents *in vivo* expansion of transfused human CD3^+^ cells

**Figure 4:**
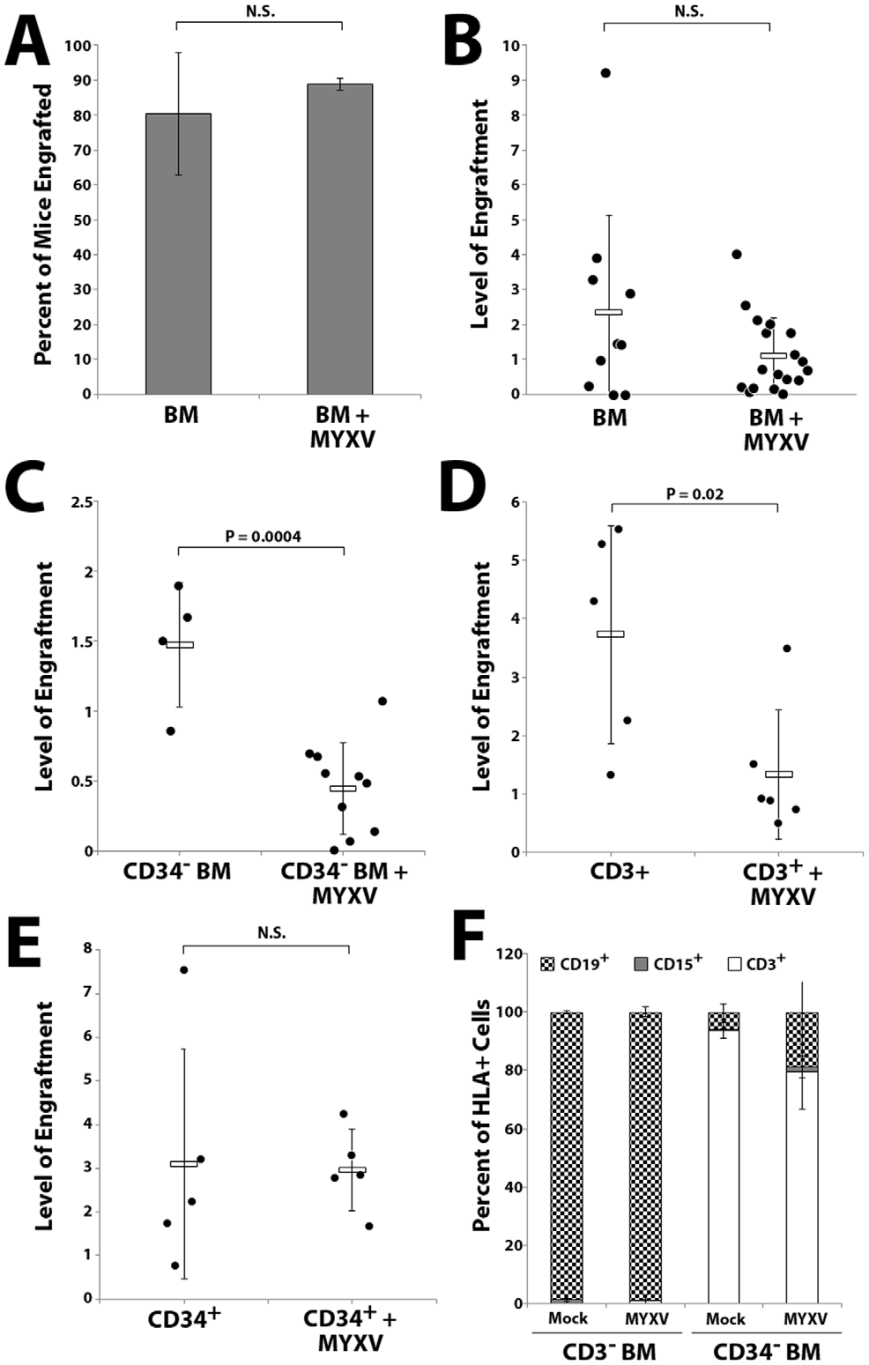
MYXV treatment prevents in vivo expansion of human donor T lymphocytes after transplant into immunodeficient mice while sparing engraftment of normal HSPCs. NSG mice were sublethally irradiated and transplanted with 1×10^7^ whole human BM cells. Six weeks after transplant, bone marrow from mice was harvested and analyzed for human hematopoietic engraftment (human CD45^+^/HLA-A, B, C^+^ double positive cells) by flow cytometry. Treatment with MYXV did not alter the proportion of animals with successful human hematopoietic engraftment (**A**) but did slightly reduce the total numbers of engrafted human cells in mouse bone marrow, which did not reach statistical significance (**B**). Irradiated mice transplanted with 1×10^7^ CD34-depleted BM (**C**) or 1×10^6^ human CD3^+^ cells (**D**) displayed lower overall levels of engraftment and this engraftment was significantly reduced by *ex vivo* MYXV-treatment. Irradiated mice transplanted with 1×10^5^ CD34^+^ selected human cells showed levels of human cell engraftment similar to those observed in mice transplanted with whole human BM. Levels of human cell engraftment were not affected by *ex vivo* MYXV-treatment (**E**). Significance was determined using Student's t-test (P<0.05). N.S  =  not significant. Lineage of engrafted cells was determined by staining HLA-A, B, C^+^ cells from murine BM with antibodies against human CD3, CD15, or CD19 (**F**).

Consistent with our previous observations [16], pretreatment of human hematopoietic stem cell grafts with MYXV did not significantly alter the proportion of mice achieving human hematopoietic cell engraftment six weeks after transplant (Figure 4A). However, mice transplanted with *ex vivo* MYXV treated whole BM did display a non-significant trend towards decreased numbers of total human repopulating cells in the BM (Figure 4B). In contrast, while all mice transplanted with CD34^+^ depleted BM or CD3^+^ selected cells also showed evidence of engrafted human cells in the bone marrow six weeks after transplant, the levels of these cells was significantly reduced by MYXV treatment (Figure 4C and 4D). Unlike the engraftment pattern of unselected BM or CD34+ selected stem cells, lineage analysis of bone marrow from mice transplanted with CD34-depleted samples revealed significant T lymphocyte skewing (Figure 4F). A similar trend was observed in mice transplanted with normal donor PBMCs, which contain CD3^+^ lymphocytes but virtually no CD34^+^ HSPCs (data not shown). In mice transplanted with *ex vivo* MYXV treated CD34^+^ HSPCs, on the other hand, we observed no reduction in the percentage of human hematopoietic cells in the bone marrow (Figure 4E) and lineage analysis revealed multi-lineage engraftment with B lymphocyte skewing typically seen xeno-transplanted immunocompromised mice (Figure 4F). These data show that *ex vivo* MYXV treatment of human HCT samples does not impair normal human HSPC engraftment in immunocompromised recipients but selectively inhibits expansion of transferred donor CD3^+^ lymphocytes in the transplant recipient.

### MYXV inhibits human:human haplo-mismatched mixed-lymphocyte-reactions

**Figure 5:**
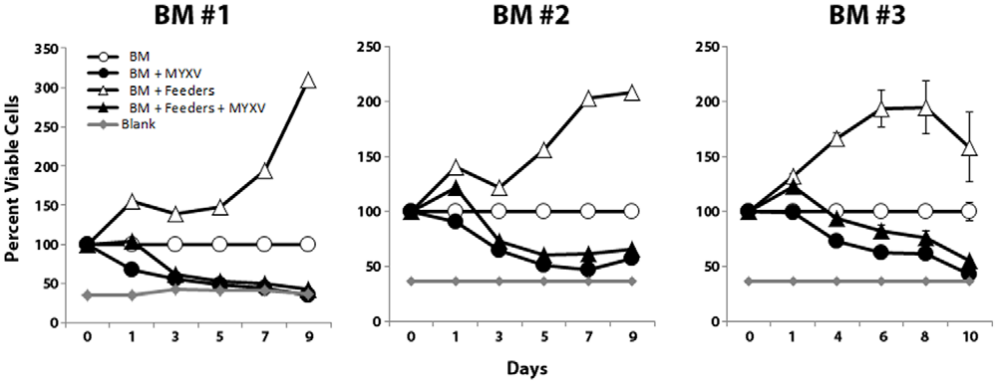
MYXV treatment prevents alloreactive human:human mixed lymphocyte expansion. To determine if MYXV inhibited expansion of allo-reactive human cells in HCT samples following human/human allo-stimulation in a mixed lymphocyte reaction assay, mock-treated or MYXV-treated human BM cells were incubated for 10 days with irradiated HLA-mismatched human feeder cells. Mock-treated BM stimulated with irradiated feeder cells showed significantly increased numbers of viable cells while MYXV-treated BM did not.

Since our *in vivo* data used a human-mouse xeno-expansion based GVHD model, we next wanted to confirm that MYXV treatment also prevented expansion of reactive cells in human:human allo-mismatched one-way mixed lymphocyte reactions (MLR). BM samples from three different donors were either mock-treated or treated with MYXV and then incubated with lethally irradiated BM feeder cells derived from an independent donor. Expansion of viable allo-reactive cells was measured at the indicated times by measuring total ATP generation using the commercial MTT assay. We found that mock-treated human BM showed a significant increase in viable cells when added to lethally irradiated HLA-mismatched human feeder cells, indicative of a typical alloreactive MLR. In contrast, pre-treatment of BM from all three test donors with MYXV prevented this MLR proliferation (Figure 5) suggesting that MYXV could also prevent expansion of allo-reactive cells from primary human BM samples when stimulated with mismatched human tissue samples.

### MYXV treatment does not abrogate beneficial graft-versus-leukemia

**Figure 6:**
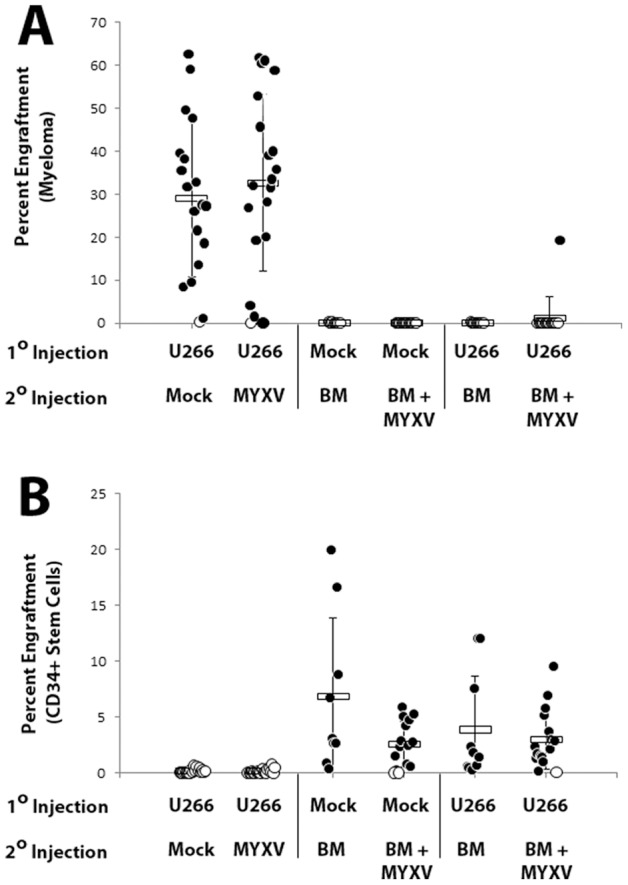
*Ex Vivo* MYXV treatment prevents GVHD while still preserving GVL. NSG mice either naïve or carrying preexisting U266 myeloma were sublethally irradiated and injected with 10×10^6^ human donor BM cells that had been either mock treated or treated with vMyx-GFP. Mice were monitored for signs of GVHD for six weeks after which BM was harvested from surviving mice and the levels of both pre-existing U266 myeloma load (**A**) and normal human HSC engraftment (**B**) were quantified using flow cytometry. Cells can be distinguished based on expression of HLA-A, B, C, HLA-A2.1 and CD45 (hematopoietic stem cell progeny are HLA-A, B, C^+^/HLA-A2.1^−^/CD45^+^ while U266 myeloma cells are HLA-A, B, C^+^/HLA-A2.1^+^/CD45^−^).

Adoptive transfer of mature CD3^+^ cells contained in alloHCT samples has been shown to improve patient prognosis through the highly beneficial GVL effect where alloreactive donor T cells engage in clearance of minimal residual disease that survived prior conditioning therapy [13], [14]. Unfortunately, the most common method for preventing GVHD, complete elimination of CD3^+^ lymphocytes, also eliminates the potential for GVL. Since MYXV treatment efficiently prevented GVHD while only directly infecting a small subset of the total CD3^+^ lymphocytes in the transplant sample, we questioned whether MYXV might selectively prevent GVHD while still permitting GVL. We therefore tested the effectiveness of the MYXV *ex vivo* purging strategy using a transplant model that measures both GVL and GVHD. Initially, non-irradiated NSG mice were injected with human multiple myeloma U266 cells and the myeloma cells were allowed to establish for two weeks. Mice were then sublethally irradiated and infused with human BM-MNCs that had either been mock-treated or treated with MYXV. Mice were observed for evidence of GVHD and mice meeting endpoint criteria were euthanized accordingly. Six weeks after injection of BM, surviving mice that had not succumbed to GVHD were euthanized and their BM analyzed for engraftment of any remaining residual myeloma as well as the expanded progeny of the HSPCs from the donor BM (Figure 6). Consistent with our previous findings, progeny of the donor HSPCs were readily observed in the BM of mice injected with normal human BM and the level of this engraftment was not significantly reduced by pretreatment of the BM with MYXV. Also consistent with our previous findings, 55% (11/20) mice transplanted with untreated human BM died from GVHD while none of the mice (0/20) transplanted with MYXV pretreatment human BM suffered any signs of GVHD. Control mice that had been pre-seeded to carry residual myeloma, sub-lethally irradiated, and then transplanted with saline showed significant remaining residual myeloma load demonstrating that the sublethal irradiation used to permit efficient BM engraftment did not clear the pre-existing myeloma niches in the mouse bone marrow. Injection of free MYXV virions alone caused no significant long term effects on existing myeloma load in the bone marrow, supporting the expectation that most of the IV-administered virus does not reach the bone marrow. Mice pre-seeded with human multiple myeloma cells, sublethally irradiated, and then transplanted with untreated BM-MNCs showed no signs of remaining cancer six weeks after transplant, indicating that the GVL effect of the donor transplant BM was highly effective at clearing pre-existing myeloma in the bone marrow in this model. Despite this beneficial effect, 50% (10/20) of mice in this cohort died from GVHD. This cohort represents the current clinical scenario of allogeneic transplant for high risk hematological malignancies. Results from this cohort also show that the presence of residual myeloma in the recipient bone marrow does not affect the development of GVHD. In the MYXV treatment cohort, only 1/20 (5%) mice showed evidence of cancer in the mouse bone marrow six weeks after injection, suggesting that *ex vivo* treatment of the human donor BM with MYXV did not inhibit the ability of this treated BM to elicit a potent GVL effect. Importantly, none of these mice (0/20) showed evidence of GVHD. Together, these results show that *ex vivo* MYXV treatment of human BM effectively prevents GVHD while still preserving beneficial GVL.

## Discussion

Previously, we demonstrated that *ex vivo* MYXV treatment prevents engraftment of primary human acute myeloid leukemia and multiple myeloma cells into NSG mice while sparing the engraftment of normal human HSPCs [16], [17]. Based on this evidence, we proposed MYXV as a novel virotherapeutic agent for purging cancer cells that contaminate autologous hematopoietic stem cell grafts [15], [25]. The data in this report present an entirely novel application of this *ex vivo* MYXV purging strategy, namely the prevention of GVHD by purging alloreactive T cells from allogeneic transplant samples, which should prove to be applicable for clinical settings such as alloHCT, donor leukocyte infusions and high-risk blood product transfusions. This is the first report of using an intact replicating virus to selectively prevent development of an allo-immune disease.

Various T lymphocyte purging methods, including positive or negative cell separations as well as treatment with specific cytotoxic agents have been previously employed to delete alloreactive T cells in order to improve alloHCT for treatment of hematologic malignancies. These methods, however, carry high risks of life-threatening infections due to delayed immunologic recovery and graft failure [13], [14]. In contrast, our data show that MYXV treatment *ex vivo* appears to have no adverse effects on normal human HSPC engraftment, which is related to the inability of this virus to bind or infect normal human CD34^+^ hematopoietic stem cells [15], [17], [18]. However, this same *ex vivo* MYXV treatment results in infection of a subset of alloreactive CD3^+^ T lymphocytes, while still allowing for the adoptive transfer of sufficient functional T lymphocytes into the recipient for beneficial GVL effects. Additionally, the *ex vivo* MYXV treatment strategy requires only a single, brief virus adsorption step prior to donor graft infusion, which could be performed with minimal changes in current good tissue practice clinical conditions (our unpublished observation and [16]). Therefore, translating this new observation into a clinical setting will not require significant deviation from the current standard of care for alloHCT, donor leukocyte infusions and high-risk blood product transfusions.

It should be noted that purging of alloreactive T lymphocytes from alloHCT samples is a fundamentally similar process to purging cancer cells from autologous hematopoietic cell grafts. Both are based on the ability of the purging agent (in this case, a virus that does not infect normal human HSPCs) to distinguish the disease-inducing cells (either contaminating cancer cells in autologous grafts or resident alloreactive donor T lymphocytes in allogeneic grafts) from the normal stem cells whose immune-reconstituting functions must be maintained (in this case CD34^+^ HSPCs). While the mechanism of MYXV's ability to discriminate alloreactive CD3^+^ T lymphocytes from HSPCs requires further investigation; our preliminary observations suggests that the safety of MYXV in terms of human hematopoietic engraftment is based on a failure of MYXV to physically bind to normal human CD34^+^ HSPCs [17], [18]. Due to the extremely broad nature of poxvirus binding for most mammalian cells [26] this suggests that MYXV might be an effective purging agent for functionally deleting a wide variety of deleterious non-stem cells from hematopoietic grafts, possibly including various classes of donor B and T lymphocytes as well as contaminating cancer cells from a wide variety of hematopoietic malignancies.

Interestingly *ex vivo* MYXV virus treatment completely abrogated CD3^+^ lymphocyte driven GVHD in 100% of xeno-transplanted animals and also prevented alloreactive human:human MLR reactions *in vitro* despite the fact that only a small, and highly variable (ranging from 2% to 25%, depending on the donor), subset of human donor CD3^+^ lymphocytes were detectably infected by MYXV, as measured by the ability to express virus-encoded EGFP. Since GVHD is highly dependent on the infused dose of donor CD3^+^ lymphocytes [27], [28], it initially appears contradictory that MYXV could block development of disease while only infecting as little as 1% of potentially disease-causing T cells. While the molecular mechanism(s) through which MYXV prevents GVHD will require further investigation, we can envision several scenarios whereby infection of only a subset of the total CD3^+^ lymphocyte population could lead to highly efficient protection from disease. For example, it is possible that MYXV might prevent GVHD by specifically infecting a minor subset of CD3^+^ lymphocytes essential for development of GVHD, such as those alloreactive T lymphocytes that actually respond to allo-antigens and proliferate in the recipient post-transplant. In a similar fashion, specific CD3^+^ subsets important for disease development, such as T_regs_, or NKT lymphocytes, could also be preferentially infected. Alternatively, MYXV might bind to a higher fraction of the total T lymphocyte population but only result in measurable GFP^+^ infection once these cells are activated, which could happen rapidly upon exposure to recipient allo-antigens following infusion. This latter hypothesis is supported by the observation that a related poxvirus, vaccinia virus, infects activated T lymphocytes at a much greater frequency than naïve T lymphocytes [23]. This explanation could also provide a potential mechanism for the ability of MYXV *ex vivo* treatment of the donor graft to prevent GVHD while sparing GVL: for example, transplant cells causing GVHD would be activated (and infected) immediately following infusion whereas cells responsible for GVL would first have to home to the murine BM before activation was initiated. Similar to what we have observed in various human myeloid leukemia cells [18], adsorption of MYXV might also inhibit T lymphocyte expansion even in the absence of fully permissive viral infection (monitored as GFP expression).

The low levels of MYXV infection observed in donor T lymphocytes are consistent with the modest levels of infection observed in several other primary hematopoietic cell types found in human BM or PBMCs, including: CD15^+^ granulocytes, CD34^+^ hematopoietic stem and progenitor cells, and platelets (data not shown). In contrast, much higher levels of MYXV infection were observed in professional antigen presenting cells, such as CD19^+^ B lymphocytes and CD14^+^ monocytes (data not shown). Therefore, it is formally possible that MYXV inhibits the development of GVHD through blocking or altering the presentation of recipient tissue antigens. However, the infusion of purified CD3^+^ lymphocytes fully reconstituted GVHD disease and MYXV treatment of just these cells was fully protective (Table S1), and thus we feel that this model is less likely.

Finally, development of GVHD in this xenograft model requires systemic irradiation prior to infusion of CD3^+^ lymphocytes (Table S1). This is similar to what is observed in human patients who have undergone myeloablative therapy and suggests that systemic inflammation might promote the development of disease. MYXV encodes several known secreted anti-inflammatory proteins that inhibit myeloid cells, such as the SERP1 glycoprotein that is currently in clinical trial to inhibit systemic inflammation associated with myocardial disease (ADD REF: Tradiff et al, 2011). Therefore, it is also possible that MYXV blocks development of GVHD through a systemic indirect mechanism.

Whether the protective effects of MYXV treatment against GVHD reported in this study are specific to treatment with MYXV, or could be duplicated using other oncolytic viruses, remains unknown. However, any other candidate oncolytic virus would also have to be fully innocuous against CD34^+^ HSPCs in order to be acceptable as a purging agent. In the case of MYXV, this safety is explained because MYXV neither binds nor infects human CD34^+^ HSPCs [16], 17.

It should also be noted that there are also a variety of autoimmune disorders caused by specific subsets of dysregulated immune cells (such as B cells, NK cells, etc) which could in theory be treated using ablative chemotherapy combined with hematopoietic transplants. However, a treatment regimen involving autologous HSPC transplantation can be complicated by disease-causing immune cells remaining in the patient autograft which can potentially mediate disease relapse. It seems likely that a virus which specifically infects the immune cell type that mediates the disease could also be used as a purging agent to remove potentially allo-reactive/disease-causing immune cells from hematopoietic transplant samples prior to infusion, thus preventing disease relapse. Additional work is obviously needed to address both these issues.

In any event, *ex vivo* MYXV virotherapy prior to infusion of allogeneic hematopoietic cells offers not only the prospect of preventing the onset of GVHD and reducing the risks of severe post-transplant GVHD, but also permits the opportunity for transplant of allogeneic grafts with greater HLA disparity such as that from mismatched unrelated donors and haploidentical donors, thereby potentially opening up alloHCT therapies to a greater number of patients with cancer or autoimmune diseases.

## Supporting Information

Figure S1 **Development of GVHD is consistently observed between various human bone marrow donors.** NSG mice were sublethally irradiated and then transplanted with 1×10^7^ human BM cells from three different donors (**A**). Mice were weighed twice per week to monitor body condition and sacrificed either six weeks post-injection or when they reached a body condition score of 2. Significant differences in survival were determined using the log-rank test (P<0.05). Post-mortem, organs were extracted, fixed in formalin, sectioned and stained for the presence of human CD3^+^ lymphocytes (**B**). Immunohistochemistry images shown are representative of results observed in five separate mice.
(TIF)

Table S1 **Development of GVHD in xeno-transplanted mice requires human CD3^+^ lymphocytes in the donor graft sample.** Sublethally irradiated NSG mice were transplanted with various human hematopoietic cell products and each cohort was observed for 6–8 weeks after transplant. Mice were sacrificed when their body condition score measured 2 (reported as death). Significance between survival of mice in the irradiated mock injected cohort and other cohorts was determined using the log-rank test (P<0.05). NS  =  not significant. N/A  =  not applicable.
(XLSX)
