# Supplementary figures and images for: Virotherapy Using Myxoma Virus Prevents Lethal Graft-versus-Host Disease following Xeno-Transplantation with Primary Human Hematopoietic Stem Cells
Source: PLoS One. 2012 Aug 14;7(8):e43298. doi: 10.1371/journal.pone.0043298 (PMC3419197; doi:10.1371/journal.pone.0043298)

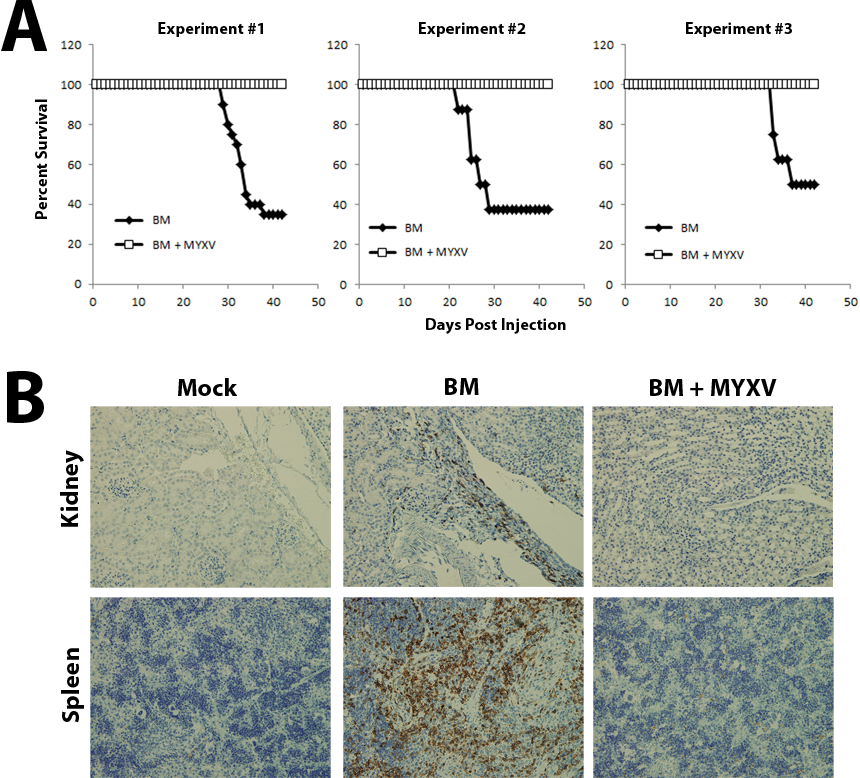

Supplement: Figure S1 — Development of GVHD is consistently observed between various human bone marrow donors. NSG mice were sublethally irradiated and then transplanted with 1×10^7^ human BM cells from three different donors (A). Mice were weighed twice per week to monitor body condition and sacrificed either six weeks post-injection or when they reached a body condition score of 2. Significant differences in survival were determined using the log-rank test (P<0.05). Post-mortem, organs were extracted, fixed in formalin, sectioned and stained for the presence of human CD3+ lymphocytes (B). Immunohistochemistry images shown are representative of results observed in five separate mice. (TIF) [file pone.0043298.s001.tif]
